# Association between COVID-19 and incidence of cardiovascular disease and all-cause mortality among patients with diabetes

**DOI:** 10.3389/fendo.2023.1230176

**Published:** 2023-07-27

**Authors:** Hee Sun Jung, Jae Woo Choi

**Affiliations:** ^1^ Big Data Department, Health Insurance Review and Assessment, Won-ju, Gangwon, Republic of Korea; ^2^ Community Care Research Center, Health Insurance Research Institute, National Health Insurance Service, Won-ju, Gangwon, Republic of Korea

**Keywords:** diabetes, COVID-19, cardiovascular disease, coronary heart disease, stroke, mortality

## Abstract

**Introduction:**

Although the risk of coronavirus disease 2019 (COVID-19) infection is higher in patients who are diagnosed with diabetes than in those who are not, research on the risk of cardiovascular disease (CVD) in COVID-19 infected patients diagnosed with diabetes compared to those who are not infected by COVID-19 is lacking. This study aimed to examine the association between COVID-19, incidence of CVD, and all-cause mortality in patients with diabetes.

**Methods:**

This study used data from the Health Insurance Review and Assessment, and included 16,779 patients with COVID-19 and 16,779 matched controls between January 2017 and June 2021. The outcomes included cardiovascular disease (CVD), coronary heart disease, stroke, and all-cause mortality. Cox proportional hazards regression models were used to evaluate these associations.

**Results:**

Patients with diabetes hospitalized because of COVID-19 had a significantly increased risk of CVD (adjusted hazard ratio [AHR], 2.12; 95% confidence interval [CI]: 1.97, 2.27) than those without COVID-19. The risks of coronary heart disease (AHR, 2.00; 95% CI: 1.85, 2.17) and stroke (AHR, 2.21; 95% CI: 1.90, 2.57) were higher in the intervention group than in the control group. In the case of all-cause mortality for middle-aged adults, we observed a higher risk in diabetes patients hospitalized due to COVID-19 than in patients without COVID-19 (AHR, 1.37; 95% CI: 1.18, 1.59).

**Conclusions:**

This study showed that patients with diabetes hospitalized due to COVID-19 had an increased risk of CVD, coronary heart disease, stroke incidence, and mortality than those who were not COVID-19 infected, suggesting more careful prevention and management among patients with COVID-19.

## Introduction

The rapid spread of coronavirus disease 2019 started from its emergence in Wuhan, China, and has affected many countries around the world, causing various complications and death in severely infected patients. In January 2020, the first case of COVID-19 was observed in Korea, with approximately 30 million patients, accounting for more than half of the total population. This is still ongoing, and the number of cumulative deaths is 34,000 approximately. Generally, COVID-19 is accompanied by clinical symptoms, including cough, fever, sore throat, and muscle pain. However, some seriously infected patients experience severe symptoms such as dyspnoea, shock, and multiple organ dysfunction syndrome (1–3). Moreover, COVID-19 has had a major effect on the incidence of severe diseases related to the cardiovascular system, including stroke, heart failure, and death (4–8).

With the ongoing COVID-19 pandemic, many studies in various nations have reported factors that increase risk of severe COVID-19 infection (9, 10). In particular, patients who have comorbidities such as hypertension or diabetes suffered from severe COVID-19 symptoms (11–15). Furthermore, the risk of COVID-19 infection is higher in patients who were diagnosed with diabetes than those who were not (16–18). These infection risk and severity of symptoms are known to be related to several clinical mechanism including the viral load due to efficient virus entry and abnormal immune system with cytokine responses (16). Therefore, it is important to investigate the risk factors linked to COVID-19 in patients with diabetes to manage the prognosis after COVID-19 infection.

Although cardiovascular disease (CVD) is a typical complication in patients with diabetes, research on the risk of CVD in COVID-19 patients with diabetes is insufficient (19, 20). In Sweden, there was a study that compared COVID-19 and influenza on the risk of severe diseases and mortality in diabetes patients (21). The risk of death was significantly higher in COVID-19 patients than in influenza patients; however, there was no statistically significant difference in stroke events. Another study compared CVD complications in COVID-19 patients with and without diabetes; however, this study included a relatively small number of participants (n = 140) registered in the hospital’s electronic medical records (22). This study showed that the risk of CVD incidence significantly increased in patients with diabetes; however, the estimation was unstable for several outcomes owing to the small sample size. However, none of these studies conducted a comparative analysis of COVID-19 uninfected patients. To understand the risk of COVID-19 infection in patients with diabetes, it is necessary to compare them with uninfected patients with diabetes as the control group.

Thus, in this study, we aimed to measure the risk of CVD incidence in COVID-19 infected diabetes patients compared with that in uninfected patients with diabetes. Furthermore, we conducted a subgroup analysis stratified by gender and age to investigate the relationship within each group.

## Methods

### Data and study sample

We designed cohort study to evaluate the risk of CVD incidence in COVID-19 infected diabetes compared with that in uninfected patients with diabetes. We used data from the Health Insurance Review and Assessment (HIRA) between January 2014 and May 2022 (23). The national health insurance system in Korea provides coverage for nearly 98% of the population, including a national medical aid program. The data includes visiting information on the medical facilities of individuals in the Korean population, such as sex, age, diagnosis, treatment, surgery, prescription, in-hospital death, and costs.

Among the 54,282,771 participants enrolled between January 2017 and June 2021, participants who did not receive a diabetes diagnosis were excluded from the study (n=46,013,518) and COVID-19 patients who were not hospitalized (n=28,232). We used the International Classification of Disease 10 (ICD-10) codes to define patients, and those who were diagnosed with diabetes (ICD-10 codes: E10-E14) more than twice within 6 months or prescribed hypoglycaemic agents were defined as patients with diabetes (n=8,268,253). Among these patients, those who were hospitalized because of COVID-19 infection were categorized as the case group (n=53,270).

**Figure 1:**
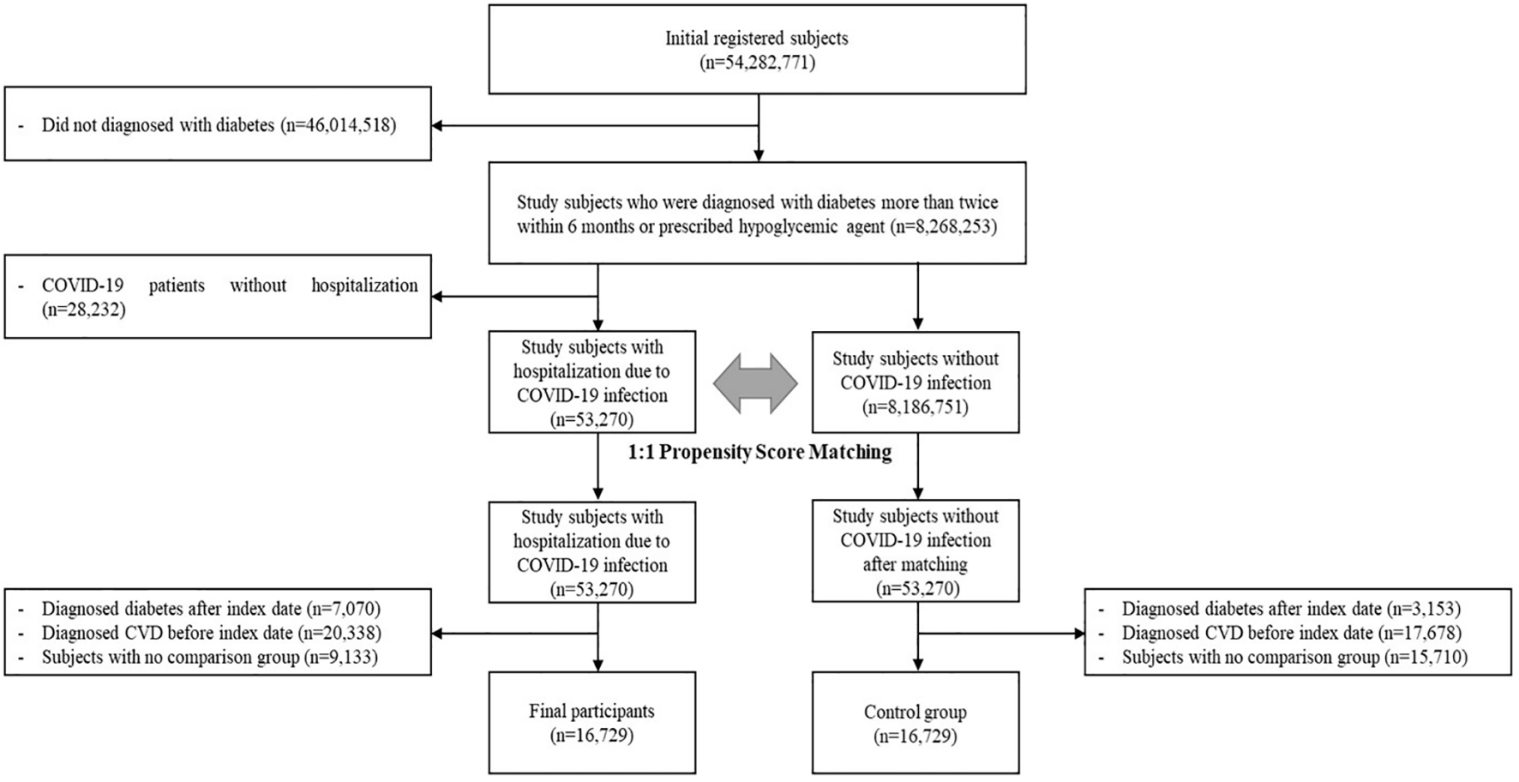
Flow chart of study participants.

For comparison, patients who had diabetes without COVID-19 infection were selected for the control group (n=8,186,751). To minimize selection bias, we performed 1:1 propensity score matching (PSM) for sex and age of individual participants. Sex and age, which were matching variables, were set based on the date of diabetes diagnosis. After completing PSM, the first date of COVID-19 infection for each individual in the case group was assigned as the index date of the matched control group. Participants were diagnosed with diabetes after the index date (n=10,223) or with CVD before the index date (n=38,016). We considered the wash-out period to be 3 years before the index date for the CVD outcome. After these exclusions, participants in the no-comparison group were excluded (n=24,843). Finally, this study enrolled 16,729 participants for each group (**Figure 1**).

### Measurements

The outcome of this study was the period from COVID-19 (ICD-10 codes: U07.1, U07.2, B34.2, and B97.2) infection to the diagnosis of CVD or date of death. We used ICD-10 codes for the diagnosis of CVD, including coronary heart disease (CHD) (ICD-10 codes: I20–I25) and stroke (ICD-10 codes: I60–I63). The period of outcomes started from the first date of COVID-19 infection between January 2017 and June 2021, which was defined as the index date. All participants were observed until May 2022 for CVD event and date of death. CVD diagnosis and death were defined as the first diagnosis or death date after the index date, respectively. For participants in whom events did not occur, period were measured by the last follow-up date.

In this study, the matched variables, sex and age, were measured based on the date of diabetes diagnosis, and the characteristics were defined as follows: the comorbidities were measured by the presence of medical history with heart failure (ICD-10 codes: I50, I11.0, I13.0, and I13.2), chronic kidney disease (ICD-10 codes: N17–N19, Z49, I12.0–I12.9, I13.1, I13.2, N08.3, E10.2, E11.2, E12.2, E13.2, E14.2, and Z99.2), peripheral artery disease (ICD-10 codes: G45, G46, I60–I69, I70.2, I73.9, I74.2–I74.9), chronic obstructive pulmonary disease (ICD-10 codes: J44), pneumonia (ICD-10 codes: J10–J18), obesity (ICD-10 codes: E66), rheumatologic disease (ICD-10 codes: M05–M14, M30–M36, and M79.0), atrial fibrillation (ICD-10 codes: I48), and cancer (ICD-10 codes: C00-C99). We observed comorbidities from the index date until 3 years ago.

### Statistical analysis

In **Table 1**, we have provided the details of demographic characteristics, such as sex and age, along with their comorbidities. All variables were compared between groups before and after matching. Number of participants and percentage are presented as categorical variables while mean ± standard deviations are presented as continuous variables. To examine the differences between groups, statistical analysis was performed using Pearson’s chi-square test and independent t-test for each type of variable.

**Table 1:** General characteristics of study participants.

Variables	Pre-PSM					Post-PSM		
	Diabetes patients with hospitalization due to COVID-19 infection		Diabetes patients without COVID-19 infection		p-value	Diabetes patients without COVID-19 infection		p-value
	N, Mean	%, SD	N, Mean	%, SD		N, Mean	%, SD	
Total	16,729		8,186,751			16,729		
Sex					<0.001			1.000
Men	8,413	50.3	4,250,051	51.9		8,413	50.3	
Women	8,316	49.7	3,936,700	48.1		8,316	49.7	
Age (mean ± standard deviation)	59.94	15.3	61.27	14.6	<0.001	60.03	15.3	0.5894
Comorbidities								
Heart failure	3,752	22.4	1,071,289	13.1	<0.001	1,317	7.9	<0.001
CKD	4,023	24.1	1,380,051	16.9	<0.001	2,839	17.0	<0.001
Peripheral artery disease	4,115	24.6	1,955,371	23.9	0.0306	3,814	22.8	<0.001
COPD	1,172	7.0	493,835	6.0	<0.001	731	4.4	<0.001
Pneumonia	6,120	36.6	1,102,796	13.5	<0.001	1,846	11.0	<0.001
Obesity	134	0.8	41,672	0.5	<0.001	113	0.7	0.1799
Rheumatologic disease	8,649	51.7	3,713,054	45.4	<0.001	7,593	45.4	<0.001
Atrial fibrillation	644	3.9	303,866	3.7	0.3459	308	1.8	<0.001
Cancer	4,228	25.3	1,278,975	15.6	<0.001	2,760	16.5	<0.001
CVD	2,944	17.6	–	–	–	1,237	7.4	<0.001
Death	1,723	10.3	–	–	–	919	5.5	<0.001

PSM, propensity score matching; COVID-19, coronavirus disease 2019; SD, standard deviation; CKD, chronic kidney disease; COPD, chronic obstructive pulmonary disease; CVD, cardiovascular disease.

For the two main outcomes, the association between hospitalization due to COVID-19 and the incidence of CVD or all-cause mortality was evaluated using Cox proportional hazards regression models. Adjusted hazard ratios (AHR) and 95% confidence intervals were calculated after adjusting for comorbidities. In addition, CVD events were separated into CHD and stroke events and evaluated for each event. First, our objective was to investigate the impact of COVID-19 hospitalization on the incidence of CVD and all-cause mortality in patients with diabetes. Second, we performed a subgroup analysis based on sex and age. Proportional hazard assumptions were assessed statistically and satisfied all the models. Statistical analysis was conducted using SAS 9.4 (SAS Institute Inc., Cary, NC, USA). This study was approved by the Institutional Review Board of Health Insurance Review and Assessment (Trial registration: 2023-018-001).

## Results

This study included 16,729 participants from the case and control groups. The average observation period was 439.0 days (standard deviation: 197.7 days). The demographic characteristics of the study participants are summarized in **Table 1**. Following 1:1 propensity score matching, there were an equal number of patients with diabetes hospitalized due to COVID-19 and in the control group (n=33,458). The mean age of the participants in each group was 59.94 years and 60.03 years, respectively, and approximately half of the participants were female (49.7%).

### Association between COVID-19 and incidence of CVD and all-cause mortality among patients with diabetes

Patients with diabetes hospitalized due to COVID-19 had a higher proportion across all categories of comorbidities. Among these comorbidities, statistically significant differences were observed between groups, except obesity. The differences were especially notable among patients with heart failure, CKD, and pneumonia. In the case group, 22.4% of participants had been diagnosed with heart failure, compared to only 7.9% in the control group. The incidences of CKD and pneumonia were 24.1% and 36.6% in the case group, and 17.0% and 11.0% in the control group, respectively.

The results of Cox proportional hazards model to examine the risk of CVD, CHD, stroke events, and all-cause mortality are summarized in **Table 2**, including the number of events, AHR, and 95% confidence interval (CI). Patients with diabetes who were hospitalized due to COVID-19 demonstrated a significantly increased risk of CVD (AHR, 2.12; 95% CI: 1.97, 2.27) than those without COVID-19. Regarding the incidence of CHD, the risk was higher in patients with diabetes who were hospitalized because of COVID-19 than in those without COVID-19 (AHR, 2.00; 95% CI: 1.85, 2.17). Furthermore, patients with diabetes who were hospitalized due to COVID-19 showed a higher risk of stroke incidence compared to those without COVID-19 (AHR, 2.21; 95% CI 1.90, 2.57). In the case of all-cause mortality, we observed a higher risk in patients with diabetes hospitalized because of COVID-19 than in patients without COVID-19 (AHR, 1.10; 95% CI: 1.01, 1.20).

**Table 2:** Association between COVID-19 and incidence of CVD and all-cause mortality among patients with diabetes .

Variables	N	Events	adjusted HR	95% CI		p-value
CVD events						
COVID-19						
No	16,729	1,237	1.00			
Yes	16,729	2,944	2.12	1.97	2.27	<0.001
CHD events						
COVID-19						
No	16,729	971	1.00			
Yes	16,729	2,256	2.00	1.85	2.17	<0.001
Stroke events						
COVID-19						
No	16,729	266	1.00			
Yes	16,729	688	2.21	1.90	2.57	<0.001
All-cause mortality						
COVID-19						
No	16,729	919	1.00			
Yes	16,729	1,723	1.10	1.01	1.20	0.030

COVID-19, coronavirus disease 2019; CVD, cardiovascular disease; HR, hazard ratio; CI, confidence interval; CHD, coronary heart disease.

### Association between COVID-19 and incidence of CVD and all-cause mortality by sex and age

**Figure 2:**
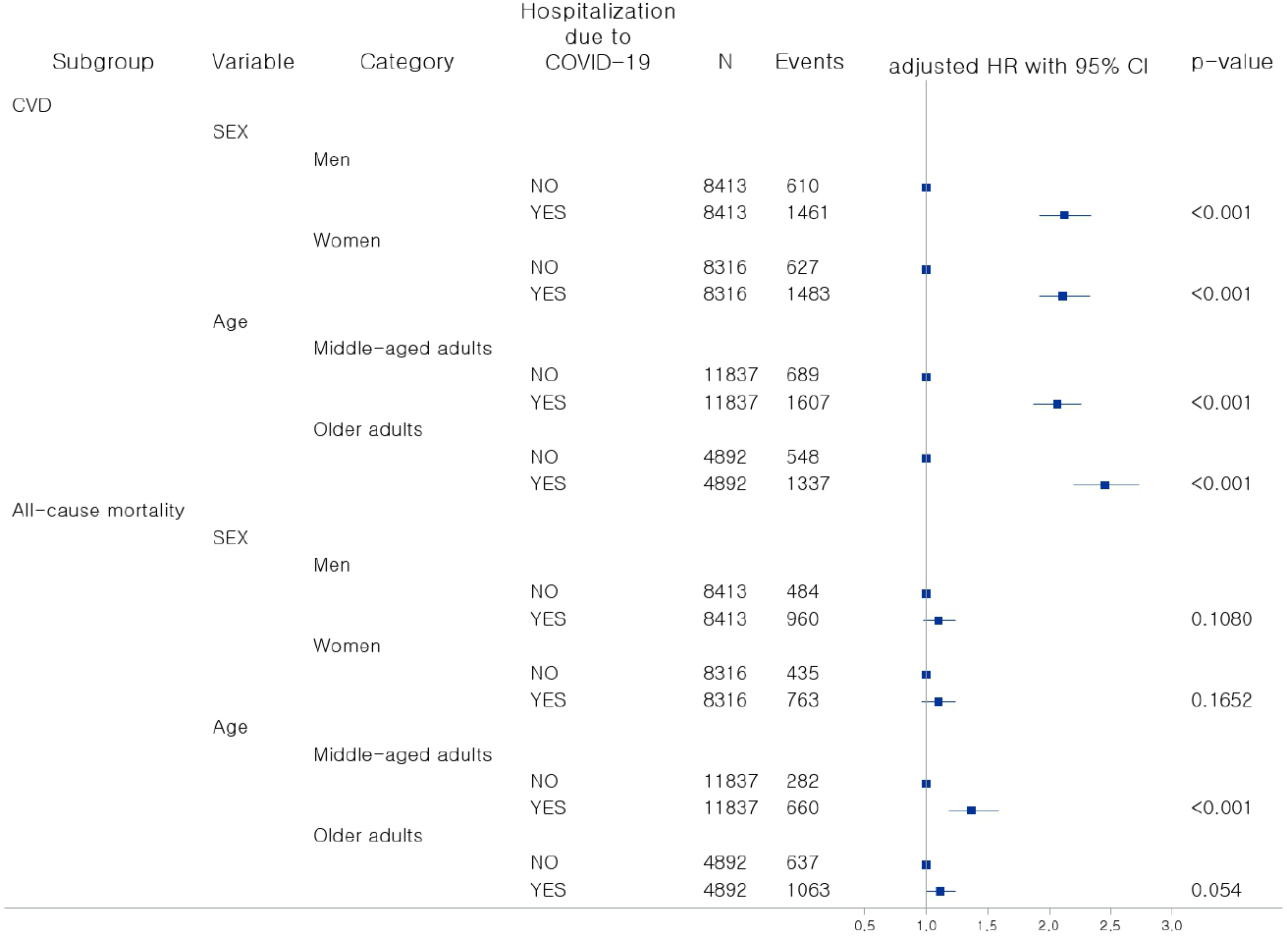
Association between COVID-19 and incidence of CVD and all-cause mortality by sex and age. COVID-19, coronavirus disease-19; HR, hazard ratio; CI, Confidence interval; CVD, cardiovascular disease.

**Figure 2** shows the results of subgroup analysis according to sex and age. For all categories of sex and age, which were men, women, middle-aged adults, and older adults, patients with diabetes hospitalized due to COVID-19 showed a significantly higher risk of CVD incidence than uninfected patients with diabetes, especially in older adults (AHR, 2.45; 95% CI: 2.20, 2.73). In terms of sex, both men (AHR, 2.12; 95% CI: 1.92, 2.34) and women (AHR, 2.11; 95% CI: 1.92, 2.33) with diabetes who were hospitalized due to COVID-19 had an increased risk for CVD incidence. For middle-aged adults with diabetes who were hospitalized due to COVID-19 had a significantly higher risk of all-cause mortality compared to those who were not infected (AHR, 1.37; 95% CI: 1.18, 1.59). There were no significant differences among the other categories.

## Discussion

In this study, we discovered that patients with diabetes who were hospitalized because of COVID-19 had an increased risk of CVD incidence and all-cause mortality, as compared to those without COVID-19. Depending on the CVD subtype, CHD incidence and stroke incidence were significantly higher in patients hospitalized due to COVID-19 infection as well. From the results of subgroup analysis according to sex and age, patients with diabetes hospitalized due to COVID-19 showed a significantly higher risk of CVD and all-cause mortality than uninfected patients for all categories of subgroups, including men, women, middle-aged adults, and older adults. The risk of all-cause mortality in the middle-aged adults was significantly higher in patients with diabetes hospitalized because of COVID-19 than uninfected patients and there was no difference in case of older adults. While the risk of death from COVID-19 infection is known to be higher in older adults, our study considered all-cause mortality due to the structure of the data. Despite adjusting for comorbidities, the risk of death from other factors remains which may explain no difference between COVID-19 infected group and uninfected group. In middle-aged adults, the risk of all-cause mortality including COVID-19 infection could be higher in COVID-19 infected group compared to uninfected group because of the risk of death from other factors is relatively low.

In a previous study, the results indicated that there was no statistically significant difference in risk of stroke and ischemic heart disease between influenza-infected patients with diabetes and patients with diabetes hospitalized due to COVID-19 for both diabetes types 1 and 2; however, the risk of death was significantly higher in patients hospitalized for COVID-19 (21). We defined the control group as patients with diabetes but without COVID-19, and we showed that the risk of CVD incidence, including CHD and stroke, was increased in patients with diabetes hospitalized due to COVID-19. These results regarding the incidence of CVD could be due to differences in the definition of the control group.

Several previous studies have examined the association between diabetes and CVD, along with the relationship between COVID-19 and CVD. In a previous study, the risk of stroke increased in COVID-19 patients, and a link between severe COVID-19 and stroke incidence was reported (4–8). These results suggest that the risk of stroke could be increased in patients with severe COVID-19 compared to uninfected individuals, especially those with known risk factors such as diabetes, which we discussed in this study. Another study that investigated the association between COVID-19 and CVD showed an increased risk of CVD, such as cerebrovascular disorders, myocarditis, and heart failure, 30 days after COVID-19 infection, indicating that follow-up management should be conducted in COVID-19 patients with cardiovascular disease (8). There are several clinical mechanism that infection of COVID-19 increase CVD disease. First, the endothelium performs various functions, including coagulation and inflammatory responses. The coronavirus can invade endothelial cells, causing direct damage to the cell membrane and thus directly impacting endothelial dysfunction. Inflammation and coagulation due to endothelial dysfunction can increase cardiovascular disease and cytokine responses due to an abnormal immune system can cause brain damage (24, 25). Second, the renin-angiotensin system, which is related to the regulation of the kidney, heart, and vascular physiology, is downregulated due to COVID-19, which can cause abnormalities in the function of organs such as the heart and brain (4–8). Third, COVID-19 infection can affect lipid metabolism. It can lead to increased levels of cholesterol and triglycerides in patients, which can induce inflammation of the vascular endothelium and increase the risk of cardiovascular disease (26).

Diabetes is known to be a risk factor for mortality in COVID-19 patients, and our study observed an increased risk of all-cause mortality among patients with diabetes who were hospitalized due to COVID-19, as compared to those who were not infected COVID-19 (11–15). This could be due to reasons such as a weakened immune system, inflammation, and control of blood sugar (11–15). From this perspective, our findings indicate that COVID-19 prevention and management are necessary to reduce mortality and CVD incidence in patients with diabetes.

This study had some limitations. First, although our data have no information on some parameters that could affect the incidence of CVD and mortality, such as body mass index (BMI), this study used the diagnosis information for obesity to adjust the effect of BMI on results (27, 28). Second, we could not control for the vaccination effect, which affects the severity of the COVID-19 infection. Although we defined the control group as hospitalized patients with COVID-19 to reflect the severity of the COVID-19 infection, the vaccination status of every study participant was unknown and this may have affected the analysis results. Third, our database was limited to the Korean population. Further studies are needed to examine our findings in other countries and ethnic groups.

## Conclusion

This study is the first to investigate the association between COVID-19 and the incidence of CVD in patients with diabetes compared to diabetes patients without COVID-19. We found that patients with diabetes hospitalized with COVID-19 had an increased risk of CVD incidence and mortality compared to those who were not COVID-19 infected. Our results indicate that careful prevention and management are needed for patients with COVID-19 diagnosed with diabetes. Further studies are required to investigate the association between COVID-19 and other severe diseases in patients with diabetes.

## Data availability statement

The datasets presented in this article are not readily available because of privacy or ethical restrictions. Requests to access the datasets should be directed to the corresponding author.

## Ethics statement

This study was approved by the Institutional Review Board of Health Insurance Review and Assessment (Trial registration: 2023-018-001).

## Author contributions

HJ and JC designed the study. HJ and JC performed the literature review and interpretation for data analysis. HJ and JC analyzed the data. HJ and JC wrote the draft. All authors contributed to the article and approved the submitted version.
